# Using Transcranial Electrical Stimulation in Audiological Practice: The Gaps to Be Filled

**DOI:** 10.3389/fnhum.2021.735561

**Published:** 2021-11-23

**Authors:** Mujda Nooristani, Thomas Augereau, Karina Moïn-Darbari, Benoit-Antoine Bacon, François Champoux

**Affiliations:** ^1^École d’Orthophonie et d’Audiologie, Université de Montréal, Montréal, QC, Canada; ^2^Centre de Recherche de l’Institut Universitaire de Gériatrie de Montréal, Montréal, QC, Canada; ^3^Department of Psychology, Carleton University, Ottawa, ON, Canada

**Keywords:** transcranial electrical stimulation, transcranial direct current stimulation, transcranial alternating current stimulation, transcranial random noise stimulation, auditory processing, auditory abilities, audiology

## Abstract

The effects of transcranial electrical stimulation (tES) approaches have been widely studied for many decades in the motor field, and are well known to have a significant and consistent impact on the rehabilitation of people with motor deficits. Consequently, it can be asked whether tES could also be an effective tool for targeting and modulating plasticity in the sensory field for therapeutic purposes. Specifically, could potentiating sensitivity at the central level with tES help to compensate for sensory loss? The present review examines evidence of the impact of tES on cortical auditory excitability and its corresponding influence on auditory processing, and in particular on hearing rehabilitation. Overall, data strongly suggest that tES approaches can be an effective tool for modulating auditory plasticity. However, its specific impact on auditory processing requires further investigation before it can be considered for therapeutic purposes. Indeed, while it is clear that electrical stimulation has an effect on cortical excitability and overall auditory abilities, the directionality of these effects is puzzling. The knowledge gaps that will need to be filled are discussed.

## Introduction

Transcranial Electrical Stimulation (tES) is a Non-Invasive Brain Stimulation (NIBS) approach, in use for several decades, that involves applying a low electrical current on the human head and assessing its effects. tES has been shown to modulate spontaneous cortical activity and excitability, leading to alterations of behavior, cognition and sensory perception (for a review: Yavari et al., 2018). tES can be generated by applying either *direct* current or *alternating* current.

*Transcranial Direct Current Stimulation* (tDCS) is the most frequently used type of tES in both clinical and research domains. tDCS has been reported to modulate resting membrane potentials by depolarizing or hyperpolarizing cortical neurons, thereby altering their firing rate (Creutzfeldt et al., 1962; Bindman et al., 1964; Purpura and McMurtry, 1965; Radman et al., 2009). This technique consists of applying a weak direct electrical current through two or more electrodes (Priori et al., 1998). The polarity of the active electrode can be positive (anode) or negative (cathode), and the effects induced by tDCS notably depend on the polarity of the current applied. Anodal tDCS (a-tDCS) at 1 mA has been shown to typically have an excitatory effect as it induces a depolarization of the resting membrane potential and consequently an increase of the firing rate of neurons (Nitsche and Paulus, 2000). On the contrary, cathodal tDCS (c-tDCS) at 1 mA induces a hyperpolarization of the resting membrane potential, which thereby decreases the firing rate of neurons. These effects were demonstrated in the motor cortex by means of Motor Evoked Potentials (MEPs); a-tDCS increased MEPs amplitude and c-tDCS decreased it (Paulus, 2011). However, a reverse effect of a-tDCS and c-tDCS can be observed depending on many factors, such as the direction of current flow relative to neuronal orientation or duration of stimulation (Jefferys, 1981; Bikson et al., 2004; Kabakov et al., 2012; Paulus et al., 2013). tDCS effects have been observed during stimulation (online effect), but also after a sufficient stimulation (offline effect). Previous studies demonstrated that the after-effect of tDCS can last for several minutes and even hours following the end of stimulation. However, to induce an offline effect, the intensity and duration of stimulation have to be adjusted. This effect is mediated by mechanisms similar to Long-Term Potentiation (LTP) and Long-Term Depression (LTD) (Bindman et al., 1964; Nitsche and Paulus, 2000; Fritsch et al., 2010; Paulus et al., 2012).

*Transcranial Alternating Current Stimulation* (tACS) consists of a sinusoidal current applied at a specific frequency that alternates between electrodes (Reed and Kadosh, 2018). This neuromodulation technique modulates neuronal firing to the external frequency applied with tACS and thereby synchronizes cortical oscillation (Antal and Paulus, 2013; Herrmann et al., 2013). tACS therefore enables to assess the influence of cortical oscillation on perception and cognition. It has been demonstrated to have an after-effect similar to tDCS, which is linked to induced neuroplastic changes (Vossen et al., 2015).

Multiple alternating currents can also be applied simultaneously in an approach known as *transcranial Random Noise Stimulation* (tRNS). tRNS induces noise by means of multiple alternating currents varying in amplitude and frequency. The bandwidth can vary from 0.1 to 640 Hz, and it can also be divided into lower (0.1–100 Hz) or higher (100–640 Hz) frequency bands. The mechanism behind the effect of tRNS is Stochastic Resonance (SR) which enables the enhancement of information processing and detection of subthreshold signals by adding noise in a non-linear system, such as the human brain (McDonnell and Ward, 2011). A previous study has demonstrated that tRNS applied on the motor cortex induces an enhancement of cortical excitability and effect lasting up to 1-h post-stimulation (Terney et al., 2008). The putative mechanism of tRNS action has been linked to the potentiation of voltage-gated sodium channels (Terney et al., 2008).

tES has been primarily shown to have an influence on motor cortical excitability by increasing and decreasing MEPs (Nitsche and Paulus, 2000), however, the application of tES on the motor cortex can also modulate a number of motor functions. Indeed, a-tDCS has been shown to improve performance of different motor tasks (e.g., Boggio et al., 2006; Vines et al., 2008; Sohn et al., 2012). Recently, there has been increasing evidence of the therapeutic effects of tES, notably an enhancement of motor functions and cognitive performances in older adults and in individuals with various neurological disorders. A recent meta-analysis reported robust beneficial evidences of a-tDCS for an array of motor functions, but also for some cognitive functions, such as working memory and language production (Summers et al., 2016).

Another clinical population that seems to benefit from tES approaches is stroke patients. Specifically, multiple studies reported that electrical stimulation, and more particularly tDCS, enhanced the upper and lower limb recovery of stroke patients showing motor dysfunctions (for review see Bai et al., 2019). Interestingly, other studies reported improvement on motor tasks as well as an enhancement of the acquisition of motor tasks in stroke patients with tDCS, and the effect lasted minutes to hours post-stimulation (Hummel et al., 2005; Zimerman et al., 2012). Stroke patients can also benefit from a-tDCS for language recovery, as such stimulation has been shown to improve naming performance and naming reaction time (Baker et al., 2010; Fridriksson et al., 2011). Furthermore, tES techniques seem to have therapeutic effects for several other neurological disorders, notably Parkinson’s disease (Broeder et al., 2015; Chen and Chen, 2019) and Alzheimer’s disease (Hsu et al., 2015; Chang et al., 2018). However, it is still unclear whether these therapeutic effects would extend to the sensory field.

Here, we review the evidence that supports the use of tES as a tool for targeting and modulating plasticity in the sensory field. Indeed, if tES is recognized as a potentiator of sensitivity at the central level, it could help to alleviate sensory loss. We more specifically sought to examine the evidence revealing the impact of tES on cortical auditory excitability and its corresponding effect on auditory processing, and in particular on hearing rehabilitation.

### The Effects of Transcranial Electrical Stimulation on Auditory Cortical Excitability

Emerging literature suggests that tES techniques can have an effect on the excitability of the auditory cortex. Indeed it was repeatedly demonstrated, as summarized in Table 1, that electrophysiological responses are modulated by electrical stimulation. Two different paradigms are generally employed to investigate the effects of tES on auditory electrophysiological responses, namely (*i*) Auditory Evoked Potentials (AEPs) and (*ii*) auditory event-related potentials (AERPs).

**TABLE 1:** Auditory electrophysiological responses.

References	Population	Paradigm	Stimulation type	Active electrode	References electrode	Stimulation parameters	Acquisition	Results
Zaehle et al. (2011)	Adults (M_*age*_ = 26) *n* = 14	Auditory evoked potentials (AEPs)	a-tDCS c-tDCS Sham	35 cm^2^ TP7 CP5	35 cm^2^ Contralateral supraorbital region	1.25 mA 0.04 mA/cm^2^ 11 min	Offline	a-tDCS (TP7) increased P50 amplitude; c-tDCS (CP5) increased N1 amplitude and a-tDCS reduced N1 latency
Chen et al. (2014)	Adults (M_*age*_ = 32) *n* = 10	Auditory ERPs: MMN	a-tDCS c-tDCS Sham	35 cm^2^ F4	35 cm^2^ Left supraorbital region	2.0 mA 0.06 mA/cm^2^ 25 min	Offline	a-tDCS reduced MMN amplitude
Heimrath et al. (2015)	Adults (M_*age*_ = 25.9) *n* = 12	Auditory ERPs: MMN	a-tDCS (HD) c-tDCS (HD) Sham (HD)	3.4 cm^2^ C5 C6	3.4 cm^2^ FC5/FC6, C3/C4, CP5/CP6, T7/T8	0.5 mA 0.1 mA/cm^2^ 21 min	Online	a-tDCS on left AC increased MMN amplitude in the temporal condition
Dunn et al. (2016)	Schizophrenia patients *n* = 36	AEPs ERPs: MMN, P3	a-tDCS c-tDCS Sham	35 cm^2^ Fp1 and Fp2	35 cm^2^ Right upper arm	1 mA 0.03 mA/cm^2^ 40 min	Offline	a-tDCS reduced MMN amplitude
Impey et al. (2016)	Adults (age: 18–35) *n* = 12	Auditory ERPs: MMN	a-tDCS c-tDCS Sham	19.4 cm^2^ C5-T7	50 cm^2^ Contralateral supraorbital region	2 mA mA/cm^2^ 20 min	Offline	a-tDCS increased MMN amplitude and c-tDCS reduced MMN amplitude in baseline-stratified groups
Heimrath et al. (2016)	Adults (M_*age*_ = 25.9) *n* = 13	AEPs	a-tDCS c-tDCS Sham	25 cm^2^ T7 and T8	50 cm^2^ Cz	1.5 mA 0.06 mA/cm^2^ 22 min	Offline	a-tDCS increased P50 amplitude in response to natural CV syllables
Rufener et al. (2017)	Adults (age: 20–35) *n* = 18	AEPs	tRNS Sham	35 cm^2^ T7 and T8		1.5 mA 0.04 mA/cm^2^ 20 min	Online	tRNS diminished P50 and N1 latency
Royal et al. (2018)	Adults (M_*age*_ = 22.6) *n* = 13	Auditory ERPs: MMN, P3	c-tDCS Sham	35 cm^2^ AF8-F8 T8-TP8	35 cm^2^ FP1-AF3-AF7	2 mA 0.1 mA/cm^2^ 20 min	Offline	c-tDCS reduced P3 amplitude
Kunzelmann et al. (2018)	Adults (M_*age*_ = 26.4) *n* = 24	AEPs	a-tDCS Sham	35 cm^2^ TP7-P7	25 cm^2^ Fp2-AF4-AF8	1 mA 0.03 mA/cm^2^ 20 min	Online and Offline	No effect of a-tDCS on auditory evoked potentials during and after stimulation
Boroda et al. (2020)	Adults (M_*age*_ = 24.9) *n* = 22	ERP-based plasticity	a-tDCS Sham	3.14 cm^2^ T7 and T8	3.14 cm^2^ Fp1 and Fp2	1 mA 0.318 mA/cm^2^ 5 min	Offline	a-tDCS enhanced N100 amplitude for the target tone, thereby it enhanced plasticity
Jones et al. (2020)	Adults (M_*age*_ = 20.9) *n* = 45	EEG during auditory click trains	40 Hz tACS a-tDCS Sham	25 cm^2^ T7	25 cm^2^ Contralateral cheek	1 mA 0.04 mA/cm^2^ 10 min	Offline	tACS increased gamma power and phase locking; tDCS enhanced the coupling of gamma activity to alpha oscillations
Hanenberg et al. (2019)	Adults (M_*age*_ = 24.3) Older adults (M_*age*_ = 70.4) *n* = 20; *n* = 19	ERP	a-tDCS c-tDCS Sham	35 cm^2^ C6-T8	98 cm^2^ Contralateral shoulder	1 mA 0.03 mA/cm^2^ 16 min	Offline	a-tDCS increased N2 amplitude in young and older adults

Zaehle et al. (2011) reported the effect of tDCS on AEPs amplitude, with a-tDCS increasing P50 amplitude and c-tDCS increasing N1 amplitude. These effects were dependent on the position of the active electrode: a-tDCS had an effect when the TP7 region was stimulated and c-tDCS had an effect when applied on CP5. Heimrath et al. (2016) demonstrated similar AEPs results in response to voiced and voiceless natural CV syllables. However, Kunzelmann et al. (2018) did not find an effect of a-tDCS on AEPs. These discrepancies can be attributed to the positioning of the active electrode and to the specificities of stimulation parameters. Indeed, Kunzelmann et al. (2018) placed the active electrode at a temporo-parietal location (over TP7 and P7), while Zaehle et al. (2011) placed the active electrode slightly higher (CP5) in the temporo-parietal area, or at a temporal level (TP7). A temporal area (T7 and T8) was also chosen by Heimrath et al. (2016), and the results were similar to Zaehle et al. (2011), as an increase of P50 was observed with a-tDCS. These results underline the importance of electrode positioning in the assessment of tES effects on electrophysiological responses. The different stimulation parameters used also contributed to the discrepancies of results; notably, current intensity, current density, the duration of stimulation and the size of electrodes were different in all three studies.

Only one study examined the effect of tRNS on AEPs. Rufener et al. (2017) revealed that such stimulation reduced P50 and N1 latency. These results are in line with the improved performance observed on the Gap Detection Task (GDT) assessed in the second part of their study (see section on “Temporal Processing”). Therefore, they suggest that tRNS can improve neural conduction time by reducing responses latencies.

Several studies also used the auditory event-related potentials paradigm to study the influence of tES on electrophysiological responses, and there are once again large discrepancies between studies. Indeed, some studies demonstrated a decrease of the mismatch negativity (MMN) amplitude with a-tDCS when stimulating frontal regions (Chen et al., 2014; Dunn et al., 2016), while others showed that a-tDCS over auditory cortices increased MMN amplitude (Heimrath et al., 2015; Impey et al., 2016). No study found effects of c-tDCS on MMN, but one reported a reduction of P3 amplitude (Royal et al., 2018). Stimulation locations and electrode montage differed between the various studies. It is expected that a variety of effects that can be obtained through different stimulation parameters, considering that MMN can originate from multiple generators.

Interestingly, Heimrath et al. (2015) used a novel type of electrode montage, namely High-Definition (HD) tDCS. This stimulation montage consists of 4 reference electrodes, making a ring around the active electrode on the target region. This electrode montage has been reported to improve spatial definition, to induce a higher electrical field and to reduce the risk of unwanted effect from a single reference electrode (Datta et al., 2009, 2012). They revealed an increase of MMN amplitude with HD a-tDCS, lateralized to the left auditory cortex.

Using an ERP-based paradigm, Boroda et al. (2020) demonstrated that a-tDCS applied bilaterally to the auditory cortex enhanced cortical plasticity. The application of a-tDCS during the repetitive presentation of the target tone induced an additional enhancement of the N100 amplitude, as compared to sham stimulation.

Another electrophysiological indication of the potential of tES approaches in rehabilitation was shown by Jones et al. (2020) who revealed that tACS and tDCS can modulate different components of auditory gamma responses. Indeed, tACS increased gamma evoked power and phase locking to the auditory stimulus, while tDCS strengthened the alpha-gamma phase-amplitude coupling in the absence of auditory stimuli. This finding is particularly interesting considering that multiple neurological disorders, such as autism spectrum disorder (Khan et al., 2013; Rojas and Wilson, 2014), schizophrenia (Edgar et al., 2014; Hirano et al., 2018) and bipolar disorder (Maharajh et al., 2007), present a disrupted auditory gamma responses and a disrupted cross-frequency coupling to gamma activity. Therefore, such tES techniques could potentially be used as a therapeutic approach on populations with these neurological disorders.

### The Effects of Transcranial Electrical Stimulation on Auditory Processing

Considering the impact of tES on auditory cortical excitability, behavioral effects would be expected to follow. Behavioral studies have focused on tES effects on temporal processing, spectral processing, binaural integration, auditory scene analysis and speech comprehension.

#### Temporal Processing

Results of different studies investigating the effect of tES approaches on temporal processing are summarized in Table 2. Using a random GDT to examine temporal resolution performance, Ladeira et al. (2011) have been the first to suggest (*i*) an enhancement of performance with a-tDCS and (*ii*) a decrease of performance with c-tDCS. Studies have also examined the effect of tES in GDT: Baltus et al. (2018) demonstrated that applying tACS at 4 Hz above the individual’s gamma frequency enabled subjects to detect significantly smaller gap sizes. On the other hand, Rufener et al. (2017) studied the effect of tRNS on the GDT, showing an enhancement of temporal processing. Conversely, Heimrath et al. (2014) showed a decrease of temporal resolution on the GDT induced by a-tDCS.

**TABLE 2:** Temporal processing.

References	Population	Paradigm	Stimulation types	Active electrode	References electrode	Stimulation parameters	Acquisition	Results
Ladeira et al. (2011)	Adults (M_*age*_ = 21.4) *n* = 11	Random gap detection task (RGDT)	a-tDCS c-tDCS Sham	35 cm^2^ T3 and T4	35 cm^2^ Right deltoid muscle	2 mA 0.06 mA/cm^2^ 10 min	Online	a-tDCS enhanced temporal resolution with 4 kHz and clicks subtests; c-tDCS reduced temporal resolution with 4 kHz subtest
Heimrath et al. (2014)	Adults (M_*age*_ = 24.4) *n* = 15	Gap detection task	a-tDCS Sham	25 cm^2^ T7 T8	50 cm^2^ C3/C4	1.5 mA 0.06 mA/cm^2^	Online	a-tDCS over left AC decreased temporal resolution
Heimrath et al. (2016)	Adults (M_*age*_ = 25.9) *n* = 13	Voice onset time (VOT) categorization	a-tDCS c-tDCS Sham	25 cm^2^ T7 and T8	50 cm^2^ Cz	1.5 mA 0.06 mA/cm^2^ 22 min	Online	c-tDCS over AC bilaterally improved categorization of CV-syllables in a VOT continuum
Rufener et al. (2016a)	Adults (M_*age*_ = 24.1) *n* = 25 Elderly (M_*age*_ = 69.8) *n* = 20	VOT categorization	tACS 6 Hz 40 Hz	35 cm^2^ T7 and T8		1.5–1.6 mA** (6 Hz) 1.3–1.4 mA ** (40 Hz) 0.04–0.05 mA/cm^2^ 8 min	Online	40 Hz tACS decreased precision of VOT categorization in adults. 40 Hz tACS enhanced precision of VOT categorization in older adults
Rufener et al. (2016b)	Adults (M_*age*_ = 25.9) *n* = 38	VOT categorization	tACS 6 Hz 40 Hz	35 cm^2^ T7 and T8		1 mA (6 Hz)** mA (40 Hz)** 0.03 mA/cm^2^ 18 min	Offline	40 Hz tACS reduced the repetition-induced improvement in phoneme categorization (reduced learning effect)
Rufener et al. (2017)	Adults (age: 20–35) *n* = 18	Gap detection task (GDT) Pitch discrimination threshold (PDT)	tRNS Sham	35 cm^2^ T7 and T8		1.5 mA 0.04 mA/cm^2^ 20 min	Online	tRNS increased detection rate for near-threshold stimuli on GDT only
Baltus et al. (2018)	Adults (M_*age*_ = 24.0) *n* = 26	GDT	tACS Individual gamma frequency (IGF) + 4 Hz IGF–4 Hz	4.9 cm^2^ FC5 and TP7/P7 FC6 and TP8/P8		1 mA 0.12 mA/cm^2^ 7 min	Online	IGF + 4 Hz tACS enhanced temporal resolution

**Intensity was adjusted to the optimal level for each participant.

The different methodologies used in these studies might explain the disparities in the effect of a-tDCS on temporal processing. First, the different results could be explained partially by the electrode montage used as Ladeira et al. (2011) used a bilateral montage that stimulated both temporal cortices simultaneously, whereas only one temporal cortex was stimulated by Heimrath et al. (2014). Furthermore, the intensity of the current applied differed between those two experiments, as well as the size of the active electrode and the position of the active and reference electrodes (extracephalic vs. cephalic). The importance of these parameters has been shown previously in the motor domain (Dissanayaka et al., 2017). For example, an extracephalic reference electrode can reduce electrical field due to the greater distance between the active and reference electrodes (Moliadze et al., 2010). However, this type of electrode montage reduces the stimulation of unsolicited cortical areas (Im et al., 2012).

To further examine the effects of tES on temporal processing, a few studies investigated the influence of tDCS and tACS on a voice onset time categorization task, with similarly conflicting outcomes. Indeed, while the results of Heimrath et al. (2016) suggest an improvement in temporal processing induced by c-tDCS, other results using 40 Hz tACS appear to be dependent of the age of the participant, indicating an improvement of performance in older subjects, and reduced performance in younger adults (Rufener et al., 2016a,b).

#### Spectral Processing

The effects of tDCS on spectral processing are summarized in Table 3. The first evidence of the impact of tDCS on spectral processing was reported by Mathys et al. (2010), as they examined the effect of a-tDCS and c-tDCS on a pitch direction discrimination task. Results showed that c-tDCS over the left and the right auditory cortex lead to reduced performance in pitch discrimination. However, a-tDCS did not have any effect on performance in this task. Other researchers also studied pitch discrimination, but with a specific focus on pitch learning (Matsushita et al., 2015). The results showed that a-tDCS blocked pitch discrimination learning, as compared to c-tDCS and sham, as the only group showing no significant improvement of threshold over the 3 days of training was the one stimulated with a-tDCS.

**TABLE 3:** Spectral processing.

References	Population	Paradigm	Stimulation type	Active electrode	Reference electrode	Stimulation parameters	Acquisition	Results
Mathys et al. (2010)	Adults (M_*age*_ = 25.9) *n* = 26	Pitch direction discrimination task	a-tDCS c-tDCS	16.3 cm^2^ C3–T3 C4–T4 O1–O2 (control)	30 cm^2^ Contralateral supraorbital region	2 mA 0.1 mA/cm^2^ 25 min	Offline	c-tDCS over the left and right AC reduced pitch discrimination
Tang and Hammond (2013) Experiment 1	Adults (age: 18–27) *n* = 15	Frequency discrimination	a-tDCS Sham	24 cm^2^ C4–T4	24 cm^2^ Contralateral supraorbital region	1 mA 0.04 mA/cm^2^ 20 min	Online	a-tDCS over right AC temporarily impaired frequency discrimination (< 24 h)
Tang and Hammond (2013) Experiment 2A	Adults (age: 18–27) *n* = 7	Frequency selectivity	a-tDCS Sham	24 cm^2^ C4–T4	24 cm^2^ Contralateral supraorbital region	1 mA 0.04 mA/cm^2^ 20 min	Online	a-tDCS reduced frequency selectivity
Tang and Hammond (2013) Experiment 2B	Adults (age: 18–27) *n* = 6	Frequency discrimination; Temporal fine structure (TFS)	a-tDCS Sham	24 cm^2^ C4–T4	24 cm^2^ Contralateral supraorbital region	1 mA 0.04 mA/cm^2^ 20 min	Online	a-tDCS decreased frequency discrimination by disrupting temporal coding
Matsushita et al. (2015)	Adults (M_*age*_ = 22.2) *n* = 42	Pitch discrimination task	a-tDCS c-tDCS Sham	35 cm^2^ Right Heschl’s gyri (HG)	35 cm^2^ Above left eyebrow	1 mA 0.03 mA/cm^2^ 20 min	Online and Offline	a-tDCS impaired auditory pitch learning
Loui et al. (2010)	Adults (M_*age*_ = 25.3) *n* = 9	Pitch perception Pitch matching Pitch production	c-tDCS Sham	16 cm^2^ TP7–C5 (STG) TP8–C6 (STG) F7–C5 (IFG) F8–C6 (IFG)	16 cm^2^ Contralateral supraorbital region	2 mA mA/cm^2^ 20 min	Offline	c-tDCS over right STG and left IFG reduced accuracy in pitch matching

*STG, Superior temporal gyrus; IFG, Inferior frontal gyrus.*

Tang and Hammond (2013) reported similar effects of tDCS on learning processes. Indeed, they showed through a series of experiments that a-tDCS had detrimental effects on frequency discrimination and learning processes, as well as on frequency selectivity. Interestingly, in their first experiment they showed that both the a-tDCS and sham groups presented a similarly rapid perceptual learning, as they both improved over the experimental blocks. Although no significant difference for rate of learning was found, subjects in the a-tDCS group were not performing as well as the sham group, suggesting a decreased frequency discrimination without affecting learning process. Nevertheless, when assessed on the second day (without stimulation), the performance of participants in the a-tDCS group nearly returned to baseline levels, while performance of the sham group remained stable. This finding suggested that a-tDCS blocked the learning and consolidation process. Furthermore, this study also suggests that tDCS had a sustained effect on frequency discrimination, as subjects in the tDCS group still performed more poorly compared to the sham group on the second day. In their second experiment, Tang and Hammond (2013) demonstrated that tDCS decreased frequency selectivity, as a-tDCS caused psychophysical tuning curves to be broader.

Pitch processing was further examined by Loui et al. (2010), using pitch perception, pitch matching and pitch production tasks. This study revealed that c-tDCS only influences pitch matching by decreasing task accuracy.

Taken together, these results suggest that a-tDCS and c-tDCS both seem to have similar effects on various spectral processing tasks, leading to a decrease in performance. However, it is noteworthy to mention that all experiments conducted so far involved healthy young adults, which were arguably already performing at an optimal level. It should be noted, also, that all experiments used a similar stimulation duration (20–25 min) and an electrode montage with an active electrode on the temporal cortex (right/left) and an extracephalic reference electrode, which may explain the homogeneous results obtained. The varying current intensity (1–2 mA), density (0.03–0.1 mA/cm^2^) and electrode sizes (16–35 cm^2^) between studies did not induce different task performance patterns.

#### Binaural Integration

The influence of tES techniques on binaural integration have only been investigated in a few experiments, as can be seen in Table 4. Using a unilateral montage, D’Anselmo et al. (2015) reported no effects of a-tDCS and c-tDCS on performance at a dichotic listening task. More recently, Prete et al. (2018) observed a similar result with unilateral high frequency tRNS (hf-tRNS). However, in a second experiment, Prete et al. (2018) demonstrated that a bilateral montage of hf-tRNS enhanced the right ear advantage. These results on the effect of tES on binaural integration suggest that only a bilateral montage can modulate this auditory ability, presumably by more efficiently inducing stochastic resonance, as compared to unilateral tRNS where only one active electrode induces noise in the system. Similarly, it was also shown in language rehabilitation and sound perception that a bilateral montage is more effective than a unilateral montage (Galletta et al., 2015; Prete et al., 2017). Differences in the parameters used might also had an impact on the results. Indeed, both studies using a unilateral montage used an extracephalic reference electrode, therefore increasing the distance between the electrodes. According to Moliadze et al. (2010) such a montage may explain the absence of a stimulation effect.

**TABLE 4:** Binaural integration.

References	Population	Paradigm	Stimulation type	Active electrode	References electrode	Stimulation parameters	Acquisition	Results
D’Anselmo et al. (2015)	Adults (M_*age*_ = 21) *n* = 47	Dichotic listening task	a-tDCS c-tDCS Sham	16.3 cm^2^ C3–T3 C4–T4	35 cm^2^ Contralateral shoulder	2 mA 0.1 mA/cm^2^ 25 min	Online	No effect of a-tDCS nor c-tDCS on dichotic listening task performance
Prete et al. (2018) Experiment 1	Adults (M_*age*_ = 22.8) *n* = 41	Dichotic listening task	Bilateral hf-tRNS Sham	25 cm^2^ 47.5 cm^2^ T3 and T4		1.5 mA 0.03–0.06 mA/cm^2^ 20 min	Online	Bilateral hf-tRNS enhanced the right ear advantage
Prete et al. (2018) Experiment 2	Adults (M_*age*_ = 24.4) *n* = 20	Dichotic listening task	Unilateral hf-tRNS Sham	25 cm^2^ T3 T4	47.5 cm^2^ Contralateral shoulder	1.5 mA 0.06 mA/cm^2^ 20 min	Online	No effect of unilateral hf-tRNS on the right ear advantage

*hf-tRNS, high frequency tRNS.*

#### Auditory Scene Analysis

Auditory scene analysis has been studied to a lesser extent in the tES domain, as can be seen in Table 5. Lewald (2016) reported no effect of tDCS on sound localization, but a significant effect on spatial sound separation. Indeed, a-tDCS placed on the left Superior Temporal Gyrus (STG) and c-tDCS placed over the right STG improved the accuracy of target localization in the left hemispace. The author suggested that bipolar tDCS with c-tDCS over the right STG increased the suppression of the activity of neuronal populations coding for locations of concurrent sounds, thus facilitating the segregation of target sound vs. concurrent sounds. This result shows the efficiency of bipolar tDCS in improving auditory segregation by decreasing concurrent neural activity. Deike et al. (2016) also revealed an effect of tDCS on auditory segregation. They used a segregation task where subjects had to listen to harmonic tone complexes and to indicate if only one stream or two separate streams were perceived. They revealed a reduction in performance following a-tDCS, which is contrary to the results of Lewald (2016). Such discrepancy can be partially related to different tasks and montage, as Lewald (2016) applied a bilateral bipolar montage and stimulated both STGs simultaneously with different current polarities, while Deike et al. (2016) used a unilateral montage with one current polarity. Different use of current intensity and density applied could also explain such differences in the results. Indeed, both stimulation parameters were higher in Deike et al. (2016). Furthermore, previous studies in the motor domain have demonstrated that increasing current intensity can invert the direction of excitability. This suggests that a-tDCS could lead to a decrease in cortical excitability, whereas c-tDCS could generate the opposite (Batsikadze et al., 2013).

**TABLE 5:** Auditory scene analysis.

References	Population	Paradigm	Stimulation type	Active electrode	References electrode	Stimulation parameters	Acquisition	Results
Lewald (2016)	Adults (M_*age*_ = 23.7) *n* = 74	Sound localization	a-tDCS c-tDCS	3.5 cm^2^ STG IPL SMC		0.4 mA 0.01 mA/cm^2^ 12 min	Online and Offline	No effect of bipolar tDCS on sound localization Left a-tDCS and right c-tDCS over STG improved spatial sound separation
Deike et al. (2016)	Adults (age: 21–41) *n* = 22	Auditory stream segregation	a-tDCS c-tDCS Sham	35 cm^2^ T7	35 cm^2^ Contralateral supraorbital region	1 mA 0.03 mA/cm^2^ 15 min	Offline	a-tDCS reduced auditory segregation
Hanenberg et al. (2019)	Young adults (M_*age*_ = 24.3) *n* = 20 Older adults (M_*age*_ = 70.4) *n* = 19	Sound localization (cocktail-party situation)	a-tDCS c-tDCS Sham	35 cm^2^ C6-T8	98 cm^2^ Contralateral shoulder	1 mA 0.03 mA/cm^2^ 16 min	Offline	a-tDCS improved localization error in young adults
Lewald (2019)	Adults (M_*age*_ = 22.6) *n* = 22.6	Sound localization (cocktail-party situation)	a-tDCS Sham	35 cm^2^ C6-T8 and C5-T7	98 cm^2^ Shoulders	1 mA 0.03 mA/cm^2^ 30 min	Offline	a-tDCS improved localization of a target speaker in a simulated cocktail-party situation

*STG, Superior temporal gyrus; IPL, Inferior parietal lobule; SMC, Somatosensory motor cortex.*

Hanenberg et al. (2019) investigated the effects of tDCS on the electrophysiological correlates of auditory selective spatial attention, with a focus on the N2 component of the ERP, in young and older adults. Their data demonstrated behavioral and electrophysiological effects of a-tDCS, more specifically an increased N2 amplitude, and improved localization performances. However, no effects of c-tDCS were revealed, in opposition to previous results (Lewald, 2016). The demonstrated impact of a-tDCS on sound localization in a cocktail-party situation in young adults was confirmed by Lewald (2019). Based on the theory that during a complex task, neuronal patterns encoding concurrent distractors are activated in addition to the patterns encoding the target stimulus, it was proposed that a-tDCS specifically increased the excitability of inhibitory interneurons that suppress irrelevant sound sources, thereby facilitating effects of selective attention.

#### Speech Comprehension

Research on the impact of tES on speech comprehension are summarized in Table 6. Giustolisi et al. (2018) demonstrated that a-tDCS over the left inferior frontal gyrus in healthy adults can improve the language comprehension of syntactically simple and complex sentences. Indeed, participants stimulated with a-tDCS for 30 min showed improved accuracy compared to participants in the sham group. Lum et al. (2019) showed that a-tDCS can have an impact on sentence comprehension, as they reported that applying electrical stimulation on the left inferior frontal gyrus improved reaction time as compared to baseline performance. Therefore, their results suggest that a-tDCS can improve the speed of sentence comprehension. On the other hand, Kadir et al. (2020) aimed at investigating whether tACS with a speech envelope could modulate speech in noise comprehension, and showed that electrical stimulation mostly worsened the speech comprehension of normal hearing adults in a background of babble noise.

**TABLE 6:** Speech comprehension.

References	Population	Paradigm	Stimulation type	Active electrode	References electrode	Stimulation parameters	Acquisition	Results
Giustolisi et al. (2018)	Adults (M_*age*_ = 22) *n* = 44	Sentence comprehension	a-tDCS Sham	9 cm^2^ F5	35 cm^2^ Contralateral supraorbital	0.75 mA 0.08 mA/cm^2^ 30 min	Online	a-tDCS over left IFG improved language comprehension of both syntactically simple and complex sentences
Lum et al. (2019)	Adults (M_*age*_ = 22.9) *n* = 36	Sentence comprehension Word comprehension	a-tDCS Sham	25 cm^2^ Between T3-Fz and F7-Cz	35 cm^2^	1 mA 0.04 mA/cm^2^ 15 min	Online	a-tDCS improved reaction time for sentence comprehension task compared to baseline performance No effect of a-tDCS on word comprehension task
Kadir et al. (2020)	Adults (M_*age*_ = 23.4) *n* = 17	Speech in noise comprehension	env-tACS Sham a-tDCS c-tDCS	35 cm^2^ T7 and T8	35 cm^2^ Cz	0.2–1.5 mA (*M* = 0.9) 0.006–0.04 mA/cm^2^ (*M* = 0.03)	Online	env-tACS modulated the comprehension of speech in noise by mostly worsening speech comprehension compared to sham

*Env-tACS, tACS with a speech envelope.*

## Discussion

The present review summarizes how transcranial electrical stimulation (tES) techniques can have a significant impact on the excitability of auditory cortical regions and its behavioral effects. Overall, research suggests that these techniques can be used to modulate nearly all auditory functions and abilities. However, there are tremendous discrepancies between studies, making it difficult to predict the directionality of the various effects.

Experimental reports on temporal and spectral processing are the only ones reporting constant outcomes. Indeed, all studies suggest that a-tDCS and c-tDCS have a positive effect on temporal processing, but a negative effect on spectral processing. Other results are scarce or often conflicting.

The results on the effect of tES techniques, notably bilateral tRNS, on binaural integration seem promising. However, more research is needed to determine whether tES could present a potential enhancing effect on binaural integration. Likewise, the potential improvement of auditory scene analysis with tDCS needs to be confirmed, as data are still preliminary. Finally, current knowledge does not allow the drawing of a clear conclusion on the impact of tES on speech comprehension, considering the very limited number of studies and the significant discrepancies between the results of these studies.

Since it is not possible to predict a constant improvement for one auditory process without affecting other aspects of auditory function, it is not possible to make any recommendation at this time. Indeed, from an audiological point of view, the use of this technique could even be harmful, and studies on spectral processing suggest that the potential applicability of tES techniques in auditory rehabilitation could be particularly damaging.

Stimulation parameters and electrode montage differ largely across studies, which could partially account for the discrepancies in the results. The importance of stimulation parameters on the induced effect has already been reported in the motor domain (e.g., Dissanayaka et al., 2017). Indeed, previous studies in the motor as well as in sensory domains demonstrate that an increase of current intensity, stimulation duration and electrode montage can invert the direction of the effect of tDCS (Paulus et al., 2013; Parkin et al., 2019). Indeed, Parkin et al. (2019) notably demonstrated that the classic effects of unilateral tDCS reported by Nitsche and Paulus (2000), as a-tDCS induces excitation and c-tDCS induces inhibition, are not extended to every stimulation protocols. No significant effect was obtained on MEP amplitude when bilateral 1 mA tDCS was applied or when the intensity was increased to 2 mA. The impact of changes in stimulation parameters are less known for tACS and tRNS, but some studies suggest that these techniques are frequency-dependent (Moreno-Duarte et al., 2014). The type of acquisition (i.e., online; offline) might also have an influence on the effect induced. Indeed, undergoing a task during stimulation could reverse the effect expected since there is ongoing brain activity due to the task (see Batsikadze et al., 2013). As such, until a concerted effort is made to use the same parameters and montage and to establish proper stimulation guidelines, there is no doubt that these observed discrepancies will persist.

Another possible explanation for the significant variations of tES results in the auditory domain could be the anatomical location of the auditory cortex, which is located deep in the superior temporal gyrus (STG) and extends to the lateral sulcus and Heschl’s Gyrus (HG). It was previously suggested that tES has a larger effect on superficial neurons (Paulus et al., 2013). This may therefore suggest that higher stimulation intensity and/or bilateral stimulation would be more likely to induce an effect on auditory functions, as it was demonstrated to stimulate deeper region in the motor cortex (Paulus et al., 2013).

Furthermore, the auditory cortex has different tonotopic gradients and neurons have different characteristic frequencies. As such, neuronal orientation may vary throughout the cortex (Talavage et al., 2004; Humphries et al., 2010; Costa et al., 2011; Langers and van Dijk, 2012; Tang and Hammond, 2013). Some studies demonstrated that the effect of tES, and more specifically tDCS, depends on the direction of the current relative to neuronal orientation. Indeed, a current applied parallel to a neuron can induce hyperpolarization while a current applied perpendicularly can induce depolarization or no effect at all (Jefferys, 1981; Bikson et al., 2004; Kabakov et al., 2012). The latter results may suggest that in the auditory cortex, where neuron orientation is not uniform, part of the neuronal population may be hyperpolarized, and another part depolarized depending on the direction of the current applied.

In the same vein, the variability of neuronal orientations in the auditory cortex underlines the importance of the active electrode position, since a small difference in position might change the effects due to a different alignment with neuronal orientation (Talavage et al., 2004; Humphries et al., 2010; Costa et al., 2011; Langers and van Dijk, 2012). This is indeed the hypothesis proposed by Ladeira et al. (2011) who showed that tDCS only had an effect at 4,000 Hz, but no effect at 500, 1,000, and 2,000 Hz. Indeed, lower frequencies are coded in the anterolateral portion of the HG, whereas higher frequencies are coded in the postero-medial part of the HG (Bhatgnagar, 2002; Langers et al., 2007). The stimulation electrodes used by Ladeira et al. (2011) were placed in the posterior portion of the temporal cortex, which may explain the specific improvement of random GDT at 4,000 Hz.

One could also suggest that it might be more appropriate to use tRNS on the auditory cortex to avoid effects of neuronal orientation, since tRNS stimulate neurons irrespective of their spatial orientation (Terney et al., 2008). As suggested by the present review, all the studies using tRNS reported an improvement in performance. However, the number of studies is too limited to conclude with certainty that tRNS is more efficient. In addition, there is no clear evidence as to which tES technique is optimal in the auditory domain. As such, the data do not support the use of this technique in audiological practice.

Another obvious shortcoming is the lack of data in populations with impaired hearing function or impaired auditory processing, notably older adults. Indeed, it is well known that a decrease in temporal processing occurs with age (Fitzgibbons and Gordon-Salant, 1996; Pichora-Fuller and Souza, 2003) and that the improvements induced by tDCS have been hypothesized to be greater in non-proficient systems (Reis et al., 2014). For example, some effects discussed earlier appear to be age-dependent—with an improved performance being observed in older subjects and conversely a reduced performance being seen in younger adults (Rufener et al., 2016a,b). These results suggest that the effects of tES on voice onset time categorization might depend on the efficiency of the auditory system. These elements alone could explain the improvements in temporal processing induced in older adults. Indeed, it is possible that tACS perturbs a normal, well-functioning system and leads to a processing deterioration in younger adults, because the neuronal reactivity level is already optimal in this group (Krause et al., 2013; Schaal et al., 2013). Future studies should therefore investigate the effect of tES techniques in older adults with hearing loss and/or impaired auditory processing, to determine if such techniques could have a clinical relevance in audiology.

Another important variable might also explain the divergence found between younger and older participants, namely, the homeostatic control of cortical excitability (Schaal et al., 2017). Unfortunately, the studies included in the present review were only conducted with normal-hearing individuals. One may therefore wonder if such variability in results would have been present in people with a suboptimal auditory system. For example, all studies using tDCS on spectral processing reported a decreased performance in the different tasks studied. However, it is important to note that all the studies reviewed studied young adults with no apparent spectral processing difficulties. Furthermore, a few studies even recruited participants with musical experience. Therefore, it can be hypothesized that the participants not only had no spectral processing impairment, but also had great auditory skills. As such, adding electrical stimulation could only disrupt that optimal level and decrease their performance. It would be important to assess the impact of tES on populations with impaired spectral processing, and then draw conclusion on the rehabilitation potential of this method.

In conclusion, the present review demonstrated that tES can have an influence on several auditory abilities. While the results are still inconclusive, the impact of the stimulation of other cortical structures on auditory perception would also deserve attention, particularly in relation to auditory scene analysis and speech comprehension, which depend on multisensory processes. Indeed, results of preliminary studies on the effect of tDCS on multisensory integration are promising. Marques et al. (2014) examined the influence of a-tDCS and c-tDCS on the McGurk illusion, a multisensory integration task. They reported that c-tDCS appears to reduce the McGurk illusion when active electrodes are placed on temporal cortices, but that applying a-tDCS to the same regions has no effect. However, a-tDCS applied on parietal cortices increased the McGurk illusion. It is therefore likely that further studies of the effect of tES on multisensory cortical structures could provide interesting avenues for audiological practice. Furthermore, this review underlined important methodological discrepancies between studies, therefore, future studies should determine the optimal stimulation parameters to apply.

## Author Contributions

MN, TA, KM-D, B-AB, and FC wrote the article. All authors discussed the results and commented on the manuscript at all stages.

## Conflict of Interest

The authors declare that the research was conducted in the absence of any commercial or financial relationships that could be construed as a potential conflict of interest.

## Publisher’s Note

All claims expressed in this article are solely those of the authors and do not necessarily represent those of their affiliated organizations, or those of the publisher, the editors and the reviewers. Any product that may be evaluated in this article, or claim that may be made by its manufacturer, is not guaranteed or endorsed by the publisher.
